# Ambulance service use by patients with lower back pain: an observational study

**DOI:** 10.29045/14784726.2022.03.6.4.11

**Published:** 2022-03-01

**Authors:** Matt Capsey, Cormac Ryan, Jenny Alexanders, Denis Martin

**Affiliations:** Teesside University ORCID iD: https://orcid.org/0000-0003-3659-5344; Teesside University ORCID iD: https://orcid.org/0000-0001-5864-4325; Teesside University ORCID iD: https://orcid.org/0000-0001-5519-3311; Teesside University

**Keywords:** adult, emergency medical services, lower back pain, prevalence

## Abstract

**Background:**

Lower back pain (LBP) is the leading cause of disability globally and can be distressing for patients. It is commonly reported that serious pathologies underlying LBP are rare and most patients would be more appropriately managed in primary care. However, recent literature suggests patients accessing emergency care may differ from those accessing primary care. Currently, little is known about the use of ambulance services by people with LBP. The aim of this study was to investigate the extent and nature of ambulance services utilisation by patients presenting with LBP.

**Methods:**

This observational study is a retrospective analysis of ambulance service calls in the North East of England presenting with LBP from 1 August 2016 to 31 July 2017 (Health Research Authority registration 17/WS/0216).

**Results:**

Of 484,495 answered calls, 3315 (0.7%) calls were categorised as initially presenting with LBP. Women represented 59% of callers. Most calls were from those aged 41–50 and 71–80 years old. Almost half of patients (48%) initially presenting with LBP were later categorised with a problem elsewhere. Of the patients, 49% received analgesia, including Entonox (24%) and morphine (13%). Most patients (69%) were transported to an emergency department while 28% remained at home.

**Conclusion:**

LBP is a relatively common reason to call the ambulance service. Contrary to data from primary care, non-spinal causes, which include medical emergencies, make up a significant proportion of this. Current guidance on back pain focuses on primary care and specialist settings. Future updates may need to consider emergency care as a distinct setting with a potentially different patient population.

## Introduction

Pressures across emergency services are increasing (Department of Health and Social Care, 2019; NHS Digital et al., 2019). Attempts to manage demand and reduce conveyance include identifying conditions that can be appropriately managed in primary care and directing individuals away from emergency services (Booker et al., 2017). The ambulance service can act as a gatekeeper to the emergency department (ED) and can refer patients back to their GP if this is more appropriate. Lower back pain (LBP) is the leading cause of disability globally and, in the main, can be suitably managed in primary care (Maher et al., 2017). The literature suggests approximately 90% of patients presenting with LBP have no objectively identifiable pathology, while about 1% have medically serious pathologies (Bardin et al., 2017; Maher et al., 2017).

There are established guidelines for the management of LBP in primary care (National Institute for Health and Care Excellence [NICE], 2020). The use of primary care guidelines in EDs has been trialled and initial reports have been positive (Tacy et al., 2017). However, other studies suggest the population of patients presenting with back pain to EDs are different to those presenting to primary care, notably in the rate of serious spinal and non-spinal pathologies (Edwards et al., 2018; Lovegrove et al., 2011; Shaw et al., 2020). LBP is a symptom rather than a condition and may or may not arise in the structures of the back (Maher et al., 2017). However, the term is also used to refer to non-specific LBP as a diagnostic category or clinical condition (Edwards et al., 2017). Commonly cited triage processes focus on serious spinal pathologies, nerve root compression and non-specific back pain (Bardin et al., 2017), but there is a further category of pain arising from elsewhere in the body (Waddell, 2004). Research conducted in EDs suggests many patients reporting the symptom LBP are eventually diagnosed with non-spinal causes including renal colic, urinary tract infections and pyelonephritis; cardiac conditions including angina and myocardial infarction; or pulmonary emboli (Lovegrove et al., 2011; Shaw et al., 2020). A recent Canadian study identified that 61% of patients presenting to the ED with LBP were diagnosed with non-specific LBP as compared to the previously cited 90% in primary care (Edwards et al., 2018). We recognise that many patients with LBP may be suitable for referral and redirection to primary care; that the ambulance service can act as a gatekeeper to ED and refer appropriate patients; and that their treatment could follow established primary care guidelines. However, the initial triage guidelines should be based on pre-hospital data to identify potential serious spinal and non-spinal conditions. At this time little is known about the extent and nature of ambulance service use by people presenting with LBP, whether they resemble those presenting to primary care or ED or if ambulance clinicians are redirecting them to alternative providers.

This observational study was a retrospective analysis of calls to the North East Ambulance Service (NEAS) categorised as presenting with ‘lower back pain’ as their primary presentation, across a 12-month period. The aim of this study was to investigate the extent and nature of ambulance services utilisation by patients presenting with LBP.

## Methods

This study analysed data collected by NEAS between 1 August 2016 and 31 July 2017. NEAS serves approximately 2.64 million people across 3230 square miles in the North East of England (Office for National Statistics [ONS], 2019). During the study period, NEAS answered 484,495 calls (NHS England, 2020). Anonymised data were analysed from all patients with the symptom impression ‘lower back pain’, assigned by the initial call taker, on their electronic patient care record (EPCR) across the 12-month period. Data included: call date and time; the patient’s age and sex; the symptom impression recorded by the call taker and the symptom discriminator impression recorded by the attending clinician; medications that were administered including dose; and patient discharge including destination if they were transported.

Initial discussions with NEAS suggested prevalence of LBP was similar to stroke. Stroke was chosen to contextualise the call volumes for LBP as it is a condition clearly recognised as a medical emergency with a recognised referral pathway. Due to practical constraints it was not possible to collect a full comparator dataset for stroke; however, prevalence comparisons were possible from the publicly reported data published by NHS England (2020).

The date and time of calls were analysed to see if there were any patterns for calls across the year, day of the week or time of day. Calls by time, day and month were measured in absolute numbers and as a percentage of the calls answered. The figures for calls answered were calculated from the ambulance service performance data published by NHS England (2020). Number of calls abandoned before answering were subtracted from the number of calls presented to the switchboard for each month. A comparison was also made with the reported numbers for ‘suspected stroke or unresolved transient ischaemic attack (TIA) patients assessed face-to-face’ in the public data.

Commonly cited triage processes for LBP focus on identifying between serious spinal pathologies, nerve root compression and non-specific back pain (Bardin et al., 2017) but, as previously identified, there is a further category of pain arising from elsewhere in the body. Symptom discriminator impression was recorded by the attending clinician from a list of available options. Fifteen separate discriminators were used for patients whose initial symptom impression had been recorded as ‘lower back pain’. These were sorted into one of three categories; those that reflected the commonly used back pain triage categories (serious spinal pathologies, nerve root compression and non-specific back pain) were categorised as ‘spinal pain’ (Bardin et al., 2017); those that reflected a condition where pain was arising from somewhere other than the lower back (categorised as ‘a problem elsewhere’); those that stated a referral destination, implying that the attending clinician was unsure of a diagnosis and was seeking a further opinion (categorised as ‘deferred diagnosis’). The dataset did not include further diagnoses made at the ED to allow comparison with the ambulance clinicians’ decision. Categorisations were agreed between two of the research team (CR and MC).

Records of medications administered were explored, including rates of medications administration and use of analgesics. Patient discharge was explored, comparing rates of those recorded as ‘see and treat’ and ‘see and convey’. Of those patients who were conveyed, choice of receiving facility was explored specifically to look for whether patients were taken to a full ED or alternative primary or urgent care options.

## Results

**Figure 1:**
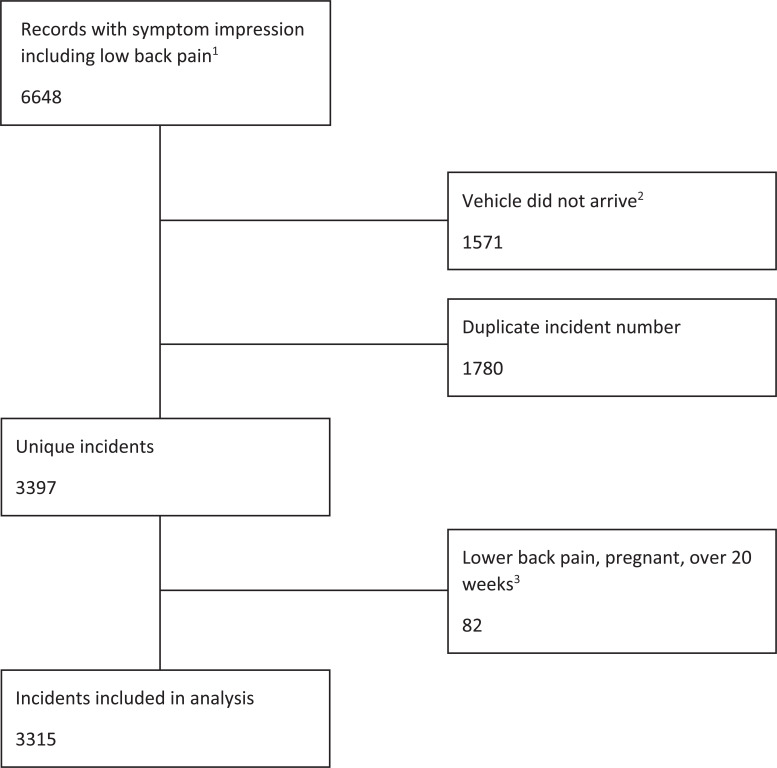
Figure 1. Data cleaning.

^1^Symptom impression is entered by the original call taker based on information given by the caller and entered into a computer-aided dispatch system.

^2^Records were produced for all vehicles assigned to an incident; however, if a higher priority incident occurred before arrival or a closer vehicle became available, the original vehicle would be stood down and have no further involvement in the incident.

^3^It was considered that in incidents recorded with a symptom impression including pregnancy, this was the presenting complaint rather than lower back pain.

Prior to data cleaning, and removal of duplicates, the dataset received from the EPCR contained 6648 entries. Multiple entries had been created for the same incident. A total of 1571 entries that contained null fields for ‘arrive scene’ time were removed. These represented occasions where a vehicle had been assigned to an incident number but subsequently stood down when another became available. Of the remaining 5177 entries, a further 1780 were removed as duplicate entries due to separate entries for medications administration. Following data cleaning, 3397 unique incidents were identified. Of the remaining incidents, a further 82 were removed as the recorded symptom impression was ‘lower back pain, pregnant, over 20 weeks’; as pregnancy was the primary condition, these incidents did not represent patients with LBP as a primary condition. This left 3315 unique incidents of patients with ‘lower back pain’ as an initial symptom impression. Figure 1 summarises the process of data cleaning.

Demographic data of patients were summarised. Callers were mostly female (n = 1965, 59.3%). Calls were highest in the age category 41–50 years, with a secondary peak at 71–80 years. This compares with ONS population estimates for the North East of England in mid-2017 suggesting that 50.9% of the population is female and that the largest age group is 21–30 years, with a secondary peak at 51–60 years (ONS, 2019).

Calls by month are summarised in Table 1. The day of week and time of day were also explored to see if there was any pattern. The highest call volume by day of week was on Sunday and Monday, and the lowest was on Friday. Calls were distributed across the day, with the peak being between 9.00 a.m. and 11.00 a.m. and a drop-off after 8.00 p.m.

**Table 1.** Lower back pain incidents as a percentage of calls received by the North East Ambulance Service switchboard by month.

	Calls presented^1^	Calls abandoned^2^	Calls answered	LBP calls (% of total answered)	Suspected stroke or unresolved TIA patients assessed face-to-face^3^ (% of total calls answered)
**Aug**	41,170	262	40,908	216 (0.53%)	382 (0.93%)
**Sept**	41,147	234	40,913	224 (0.55%)	317 (0.77%)
**Oct**	43,381	166	43,215	268 (0.62%)	297 (0.69%)
**Nov**	40,184	182	40,002	273 (0.68%)	298 (0.75%)
**Dec**	44,972	251	44,721	241 (0.54%)	257 (0.57%)
**Jan**	41,731	226	41,505	251 (0.60%)	317 (0.76%)
**Feb**	37,085	127	36,958	221 (0.60%)	273 (0.74%)
**Mar**	37,698	125	37,573	316 (0.84%)	305 (0.81%)
**Apr**	36,254	205	36,049	337 (0.93%)	322 (0.90%)
**May**	41,577	331	41,246	321 (0.78%)	322 (0.78%)
**Jun**	39,594	159	39,435	325 (0.82%)	315 (0.80%)
**Jul**	42,228	258	41,970	322 (0.77%)	285 (0.68%)
**Total**	487,021	2526	484,495	3315 (0.68%)	3690 (0.76%)

^1^Number of calls presented to the ambulance service switchboard from 999; ^2^number of calls abandoned before answering, taken from the publicly reported ambulance service data; ^3^category taken from the publicly reported ambulance service data.

LBP = lower back pain; TIA = transient ischaemic attack.

The categorisation of symptom discriminator impression, as recorded by the attending clinician from a list of available options, is shown in Table 2. A total of 47.9% (n = 1587) of callers were categorised as pain arising from a problem elsewhere, of which the majority of sub-conditions could be considered medical emergencies. The symptom discriminator impressions that were categorised as representing spinal pain were limited and almost all were recorded as spinal cord compression (n = 1151; 34.7%). The only other symptom discriminator impression recorded that reflected spinal pain was ‘spinal injury’, although only one call was recorded as a spinal injury. This low number suggests that patients who had LBP as a symptom of a spinal injury (or other trauma cause) will have been initially recorded by the call taker under a symptom impression other than ‘lower back pain’. In the deferred diagnosis category, 14.2% (n = 471) of patients had a symptom discriminator impression that related to the requirement for referral for further assessment and management.

**Table 2.** Categorisation by symptom discriminator impression.

A problem elsewhere^1^		Spinal pain^2^		Deferred diagnosis^3^	
Acute abdomen	535 (16.1%)	Spinal cord compression	1151 (34.7%)	Full primary care assessment and prescribing capability	397 (12.0%)
Ischaemia, non-trauma	296 (8.9%)	Spinal injury^4^	1 (< 0.1%)	Full ED assessment and management capability	74 (2.2%)
Aortic aneurysm, rupture/dissection	231 (7.0%)			Ambulance dispatch	2 (< 0.1%)
Ectopic pregnancy	213 (6.4%)			Not recorded	103 (3.1%)
Septicaemia	117 (3.5%)				
Deep vein thrombosis	144 (3.4%)				
Gastrointestinal bleed	48 (1.4%)				
Acute coronary syndrome	1 (< 0.1%)				
Toxic ingestion	2 (< 0.1%)				
	1587 (47.9%)		1152 (34.8%)		576 (17.4%)

The symptom discriminator impression is selected from a large pre-determined list contained within the electronic patient record system. The table shows the 15 symptom discriminator impressions that ambulance clinicians used to further categorise calls received that had been listed as lower back pain following a face-to-face assessment.

^1^Categorised as patients where the lower back pain was caused by a problem occurring somewhere other than the spine. ^2^Categorised as patients where the lower back pain was caused by a spinal pathology; the number of symptom discriminator impressions available is very limited and it is not possible to tell how clinicians made their diagnosis. ^3^Discriminator impressions that did not refer to a condition and includes patients where clinicians were unable to make a diagnosis. ^4^Only one call initially categorised as lower back pain was subsequently categorised by the attending clinician as spinal injury; other patients with spinal injury will have called the ambulance service but these will have been categorised differently.

ED = emergency department.

A range of medications had been recorded in the electronic patient record. A total of 1787 (53.9%) received pharmacological treatment of some type; 1618 (48.8%) received analgesia. The most frequently used was Entonox (n = 804, 24.3%), followed by morphine (n = 430, 13.0%). Of the patients, 281 (8.5%) received paracetamol, 78 (2.4%) received ibuprofen and the remaining 55 received other analgesics (co-codamol, codeine, diclophenac and ketamine). There were 34 patients who received diazepam; 32 of these received oral diazepam given under Patient Group Direction for ‘treatment of acute lower back pain due to paraspinal muscle spasm’.

Patient discharge and destination hospital were recorded in the electronic patient record. A total of 902 (27.2%) were recorded as ‘see and treat’ in the home setting. The majority were transported to an ED (n = 2297, 69.3%). The remaining 112 (3.4%) went to a variety of different locations including: health centre, hospital ward, major trauma unit, minor injury unit, primary percutaneous coronary intervention and walk-in centre.

## Discussion

The aim of this study was to investigate the extent and nature of ambulance services utilisation by patients presenting with LBP. A small body of research has explored the use of emergency services by patients with LBP but with little focus on emergency ambulance services (Edwards et al., 2018). Only one paper looking at ambulance use is reported in the literature (Phillips et al., 2012), which was carried out in Barbados and found that 0.9% of calls were for ‘back pain (non-traumatic)’, close to the current study’s figure of 0.68%. The data suggest calls in this group are similar in number for stroke and TIA. This demonstrates that while the percentage of calls is small in absolute terms it is comparable to another condition with a recognised referral pathway. Similar focused care, triage guidance and pathways for LBP could make an impact on patient care and ambulance service workload.

No clear monthly pattern was discernible. Calls were distributed across the day, with the peak being between 9.00 a.m. and 11.00 a.m. and a drop-off after 8.00 p.m. The highest call volume by day of week was on Sunday and Monday, and the lowest was on Friday. It was considered possible that LBP-related calls to ambulance services would increase when primary care facilities were closed; however, the current data do not show an increase in calls during out-of-hours periods. This would support the argument that those people accessing the ambulance service with LBP are a different group to those accessing primary care. It also aligns with research that suggests patient decision making is not based on the availability of services but on what they are perceived to offer (Booker et al., 2017; O’Cathain et al., 2020).

Much of the literature suggests LBP is suitable for management in primary care, with very few patients having underlying medical conditions requiring emergency care. Maher et al. (2017) suggest that about 90% of patients with LBP have non-specific LBP defined by having no clear pathological cause. This is a value commonly quoted within the LBP literature. However, Edwards et al. (2018) in their study of a Canadian ED found 22.5% of patients presenting with LBP as a symptom had a final diagnosis other than LBP. The data from our study support this more complex situation within the ambulance service setting. Nearly half of patients (47.9%) in this study were categorised by the attending clinician as having a cause arising from elsewhere in the body, including serious pathologies such as dissecting aortic aneurism and ectopic pregnancy. The LBP literature rarely mentions this sub-group of patients. The low incidence of serious or underlying conditions (1%) predominantly refers to those arising in the back or spine, such as cauda equina syndrome, spinal cancer or conditions such as ankylosing spondylitis, rather than serious pathologies elsewhere in the body that are associated with pain in the lower back. Accurate identification of these serious non-spinal pathologies by clinicians in emergency care settings (pre-hospital and ED) may contribute to the low numbers seen in primary and secondary care. This apparent, considerable difference in patient make-up between those with back pain presenting to emergency care compared to primary care suggests that different populations may be presenting to both services. As such, directly introducing primary care guidelines into the emergency setting may not be appropriate. Any guidance for ambulance clinicians should take account of the increased likelihood of encountering serious, non-spinal pathologies.

The largest diagnosis sub-category overall was spinal cord compression (n = 1151, 34.7%). Spinal cord compression usually arises as a complication of trauma or metastatic cancer. NICE (2008) estimates the incidence of metastatic spinal cord compression in England and Wales to be around 80 cases per million people every year. This would equate to about 210 cases annually in the North East region. Spinal cord compression due to trauma is unlikely to have been recorded as ‘lower back pain’ by the initial call taker. It is not clear from the data how ambulance clinicians made this diagnosis as the available options for final diagnosis were limited to a small set of options, for example there was no category for nerve root compression. Of the patients who were recorded as having ‘spinal cord compression’, about a quarter (n = 296) were not transported and were recorded as ‘see and treat’ under patient disposal. This suggests the discriminator impression ‘spinal cord compression’ may be being used for a wider range of patients than the term suggests. Ambulance service clinical guidelines focus on spinal cord injuries (Association of Ambulance Chief Executives & Joint Royal Colleges Ambulance Liaison Committee, 2019) and there is little in the educational literature targeting paramedics on the broader topic of back pain beyond managing acute trauma (Nicholls & Hawkes-Frost, 2012). This does not exclude paramedics developing their knowledge from other sources. Further explanation may have been present in free text notes, but these were not available for this study.

Current guidance on the treatment of lower back pain and sciatica (NICE, 2020) does not advocate the use of opioid analgesics or diazepam. However, these medications were used by ambulance clinicians in the treatment of patients. Morphine was the second most common drug administered (n = 340) after Entonox. The stark difference between NICE guidance and ambulance service practice likely reflects the primary/secondary/tertiary care focus of the guidelines and identifies an important gap in NICE guidelines regarding emergency service care. NICE advocates oral NSAIDs rather than opioids or paracetamol; however paracetamol has been shown to be effective in the immediate to short term (Saragiotto et al., 2016). Of the callers, 340 (13%) received morphine while 281 (8.5%) received paracetamol and only 78 (2.4%) received ibuprofen. The most commonly used analgesia was Entonox, with 804 (24.3%) callers receiving it. Its absence from NICE guidance on managing people presenting with LBP further suggests that the guidance is not written for presentations to emergency care. This identifies an important gap in the evidence for front-line clinicians.

While LBP is usually considered suitable for management in primary care, of the patients seen in this study 2297 (69.3%) were transported to an ED. This appears similar to the conveyance rate for all incidents receiving a face-to-face response across the trust, which was 64.0% in the study period (NHS England, 2020). Of the 3.0% of patients who were conveyed to somewhere other than an ED, there was a range of facilities including walk-in centres, urgent care centres and direct to a ward. This provides some limited evidence that where alternative referral options exist, some ambulance clinicians are willing to use them.

### Limitations

Limitations of this study include its retrospective observational design, thus no claims of cause and effect can be made. That these data were generated within real-life clinical practice is a strength; however, it also introduces limitations. It was not clear if missing data were due to omissions or indicated that something had not occurred (e.g. the pain score data). The limited options available for clinicians to record their impressions mean that categories for the symptom discriminator impressions were broad. Care should be taken with these clinician-assigned categorisations as, in addition to being drawn from a pre-determined list, they have not been compared to final diagnoses. The LBP literature usually uses further diagnostic categories; however, it was felt that the categories used in this study are too broad or ill-defined for further analysis, making comparison with the LBP literature difficult.

## Conclusion

These data suggest that LBP makes up a similar number of calls to ambulance services to stroke. Primary care guidance suggests patients with LBP can be safely managed in primary care and there are international projects to introduce new pathways of care, such as physiotherapists, into EDs to manage those who do present (Machado et al., 2018; Sayer et al., 2018). However, generalising guidelines from one population to another may not be appropriate. This study suggests that the population of people with LBP presenting to ambulance services may be similar to those presenting to ED and different to those presenting at primary care services. However, it is not possible to confirm this without further studies following patients through to a final diagnosis. There may be a gap in clinical guidance for how patients with LBP presenting to ambulance services should be managed.

## Acknowledgements

We would like to thank the North East Ambulance Service NHS Trust for their support in providing the data for this article.

## Author contributions

All authors have contributed substantially to the manuscript in keeping with ICMJE authorship guidelines. MC acts as the guarantor for this article.

## Conflict of interest

None declared.

## Ethics

The study received ethical approval from the Health Research Authority (REC ref: 17/WS/0216). The study protocol was registered with Clinicaltrials.gov (ID: NCT03474068).

## Funding

None.
